# Sequencing of the variable region of *rpsB* to discriminate between *Streptococcus pneumoniae* and other streptococcal species

**DOI:** 10.1098/rsob.170074

**Published:** 2017-09-20

**Authors:** Anne L. Wyllie, Yvonne Pannekoek, Sandra Bovenkerk, Jody van Engelsdorp Gastelaars, Bart Ferwerda, Diederik van de Beek, Elisabeth A. M. Sanders, Krzysztof Trzciński, Arie van der Ende

**Affiliations:** 1 Department of Paediatric Immunology and Infectious Diseases, Wilhelmina Children's Hospital, University Medical Center Utrecht, Utrecht, the Netherlands; 2 Department of Medical Microbiology, Academic Medical Center, Amsterdam, the Netherlands; 3 The Netherlands Reference Laboratory for Bacterial Meningitis, Academic Medical Center, Amsterdam, the Netherlands; 4 Department of Neurology, Academic Medical Center, Amsterdam Neuroscience, Amsterdam, the Netherlands; 5 Centre for Infectious Disease Control, National Institute for Public Health and the Environment (RIVM), Bilthoven, the Netherlands

**Keywords:** mitis group streptococci, *Streptococcus pneumoniae*, carriage, invasive disease, ribosomal S2-typing

## Abstract

The vast majority of streptococci colonizing the human upper respiratory tract are commensals, only sporadically implicated in disease. Of these, the most pathogenic is Mitis group member, *Streptococcus pneumoniae*. Phenotypic and genetic similarities between streptococci can cause difficulties in species identification. Using ribosomal S2-gene sequences extracted from whole-genome sequences published from 501 streptococci, we developed a method to identify streptococcal species. We validated this method on non-pneumococcal isolates cultured from cases of severe streptococcal disease (*n* = 101) and from carriage (*n* = 103), and on non-typeable pneumococci from asymptomatic individuals (*n* = 17) and on whole-genome sequences of 1157 pneumococcal isolates from meningitis in the Netherlands. Following this, we tested 221 streptococcal isolates in molecular assays originally assumed specific for *S. pneumoniae*, targeting *cpsA*, *lytA*, *piaB*, *ply*, Spn9802, *zmpC* and capsule-type-specific genes. Cluster analysis of S2-sequences showed grouping according to species in line with published phylogenies of streptococcal core genomes. S2-typing convincingly distinguished pneumococci from non-pneumococcal species (99.2% sensitivity, 100% specificity). Molecular assays targeting regions of *lytA* and *piaB* were 100% specific for *S. pneumoniae*, whereas assays targeting *cpsA*, *ply*, Spn9802, *zmpC* and selected serotype-specific assays (but not capsular sequence typing) showed a lack of specificity. False positive results were over-represented in species associated with carriage, although no particular confounding signal was unique for carriage isolates.

## 1. Background

Viridans streptococci are Gram-positive bacteria, many of which have evolved alongside the human host as commensals of the upper airways and oral cavity [1,2]. Interspecies genetic recombination has played a large role in their evolution [1,3] and often makes taxonomical classification a difficult task [4]. Streptococci of the Mitis group [5] present particular challenges as phylogenetic studies report tight associations within the group, reflected by multiple evolutionary lineages with boundaries that are hard to define [2,5–7]. The exception to this, comprising a single evolutionary lineage, is *Streptococcus pneumoniae*—considered to be the most pathogenic of all Mitis group members [2,5,8].

Asymptomatic colonization of the upper respiratory tract by *S. pneumoniae* is considered prerequisite for disease, of which the most severe forms are meningitis and bacteraemia with or without pneumonia, collectively described as invasive pneumococcal disease (IPD). The lower pathogenic potential of other Mitis group members is reflected by smaller genomes relative to *S. pneumoniae* [6]. This is likely the result of a reductive evolutionary process leading to the loss of virulence genes [6], which in turn increases genome stability [1,6]. The most important virulence factor of *S. pneumoniae* is considered to be its polysaccharide capsule; unencapsulated (non-typeable) strains seldom cause IPD [9,10]. The genomic flexibility of pneumococci has assisted in the great antigenic diversity of the capsular polysaccharides, with evidence of capsular genes imported not only from Mitis group species but also from more distant groups of Anginosus and Salivarius streptococci [2,11]. This has resulted in the classification of over 90 pneumococcal serotypes [12]. To date, the capsule remains the only target for currently marketed pneumococcal vaccines. However, conjugated polysaccharide vaccines (PCVs; protective in all ages) target a maximum of 13 serotypes [13]. Following vaccine introduction, surveillance of pneumococcal disease and carriage have been important measures of direct and indirect effects of vaccination on serotype distribution [14].

The gold standard method for pneumococcal detection is conventional diagnostic culture [15] which relies on colony morphology, sensitivity to optochin and solubility in bile salts. However, some streptococci generate atypical reactions in these assays [1,6,16–20], requiring additional biochemical, serological or genetic tests for species determination [21]. Following the identification of a strain as *S. pneumoniae*, serotype is usually determined by the capsule swelling (Quellung) [22] or co-agglutination methods [23].

Culture-independent, molecular diagnostic methods of pneumococcal detection are reported to be of higher sensitivity as compared to conventional culture [24–26]. In carriage surveillance, the sensitivity of *S. pneumoniae* and pneumococcal serotype molecular detection can be further increased by sampling the oral niche in addition to the standard nasopharyngeal swab [15,25,27–30]. However, the high microbial diversity in the oropharynx and saliva [31] is reflected by a greater abundance of other streptococci, carrying homologues of pneumococcal genes [1,3,11,32] and increasing the risk of non-specific results [33,34]. This has become evident throughout the evolution of molecular assays developed for the discrimination and detection of *S. pneumoniae*, exemplified by assays targeting genes *ply* (encoding pneumolysin) [35] and *lytA* (encoding the major autolysin) [36] and DNA fragment Spn9802 (unknown function) [37]. Despite high sensitivity all initial assays proved to be lacking in specificity, subject to confounding by close relatives including *Streptococcus pseudopneumoniae* and *Streptococcus mitis* [17,19,35,38–40]. The development of quantitative-PCR (qPCR) overcame this limitation for *lytA* [35], which is now widely accepted as the molecular determinant of pneumococci, proving both highly sensitive and specific [19,27,29,35,39–41]. Owing to the challenges in achieving both high sensitivity and specificity in molecular assays developed by others, *S. pneumoniae* gene *piaB* (encoding the iron acquisition ABC transporter lipoprotein PiaB) has gained our interest [27,42]. With *piaB* never being detected in oral streptococci, including *S. mitis* isolates known to possess *ply* and *lytA* [16,43], and with the protein being 100% conserved between pneumococcal isolates, it has been suggested that PiaB is unable to evolve through the process of horizontal DNA transfer (HDT) and thus is unlikely to be transferred to species related to *S. pneumoniae* [16].

In this study, we introduce a new molecular method for the identification of streptococcal species, based on ribosomal multilocus sequence typing (rMLST), developed by Jolley *et al.* [44] for bacterial strain classification. Of the 53 ribosomal protein (*rps*) genes analysed in rMLST, we identified a region in *rpsB* (a single-copy gene encoding the 30S ribosomal protein S2) which could potentially discern species of streptococci. We validated this new method on over 200 streptococcal strains cultured from patients with severe streptococcal disease and from asymptomatic carriage and used this collection to further assess the specificity of molecular assays designed by us [27] and others [26,29,38,45–50] to detect pneumococcal gene sequences (including serotype-specific sequences and sequences encoding potential virulence factors) in clinical samples. We selected these assays primarily based on their previous application in diagnostic settings [26,29,38,45–50]. In the case of assays for which false positivity was already reported [24,28,34,42], we aimed to identify non-pneumococcal species responsible for confounding. We found that only those assays targeting *S. pneumoniae*-unique sequences within the genes of *lytA* and *piaB* were fully specific for pneumococci. We identified species which may confound diagnostic methods of *S. pneumoniae* and pneumococcal serotype detection. These findings stress the importance of critical interpretation of results from genotypic tests used both in clinical settings and in epidemiological surveillance on carriage and disease.

## 2. Methods

### 2.1. Study isolates

Streptococcal strains were isolated from patients with streptococcal disease and asymptomatic carriers (table 1). From disease, we selected 101 non-pneumococcal streptococcal strains received by the Netherlands Reference Laboratory for Bacterial Meningitis (RLBM) between 2000 and 2015. Of these, 70 were isolated from cerebrospinal fluid (CSF), 24 from blood, three from sputa and one isolate each from a wound, joint puncture, bronchoalveolar lavage and an unrecorded sample type. We included all α-haemolytic strains and a maximum of 10 isolates per species of β-haemolytic streptococci received by RLBM in this period. From asymptomatic carriage, one α-haemolytic, catalase-negative colony was selected per culture per individual. In total, 103 strains were isolated from saliva of older adults (*n* = 51, greater than or equal to 60 years old) and from nasopharyngeal samples from children (*n* = 52, less than or equal to 2 years old). We also included 17 strains classified upon isolation in previous carriage studies [24,27] as optochin-sensitive yet Quellung non-typeable [15], thus unencapsulated, *S. pneumoniae*. In addition, nine strains of non-streptococcal species common in the upper respiratory tract, one each of *Bacteroides fragilis, Haemophilus influenzae, Neisseria meningitidis, Klebsiella oxytoca, Klebsiella pneumoniae, Pseudomonas aeruginosa, Staphylococcus epidermidis, Staphylococcus aureus* and *Moraxella catarrhalis*, were also included.

**Table 1.** Overview of streptococcal strains included in the study (n.a., demographic data not available).

strain type	source	n	infant	adult	CSF^b^	blood	sputa	other^c^	NP^d^	OP^e^/saliva
non-pneumococcal^a^	disease	101	n.a.	n.a.	70	24	3	4	—	—
	carriage	103	52	51	—	—	—	—	53	50
NT^f^ pneumococci	carriage	17	12	5	—	—	—	—	17	—
overall		221	64	56	70	24	3	4	70	50

^a^Strains annotated based on sequence analysis of the ribosomal S2 gene.

^b^Cerebrospinal fluid.

^c^Wound, joint puncture, bronchoalveolar lavage fluid, unknown.

^d^Nasopharynx.

^e^Oropharynx.

^f^NT, non-typeable (presumably acapsular) pneumococci.

### 2.2. Bacterial DNA extraction

Columbia agar plates supplemented with 5% sheep blood were inoculated from a frozen stock of a single-colony passed culture of a strain and incubated overnight (37°C, 5% CO_2_). DNA was extracted from plate growth harvests using the DNeasy Tissue kit (QIAGEN, Venlo, the Netherlands). Template DNA concentration was determined by 16S-based real-time qPCR [31].

### 2.3. Streptococcal species identification (S2-typing)

Streptococcal S2-typing was designed analogous to the method for the identification of *Neisseria* species [51]. Briefly, genes encoding streptococcal ribosomal proteins were extracted from the NCBI database (https://www.ncbi.nlm.nih.gov). Potentially suitable genes were selected based on their length, variability among streptococci, the ability to discriminate between streptococcal species among a limited set of strains and the presence of conserved sequences with a space of approximately 400 base pairs, appropriate for designing PCR primers. Eventually, *rpsB* encoding ribosomal protein S2 was selected and primers S2F (5′-ATGGCAGTAATTTCAATG-3′) and S2R (5′-GAATTTTTCAAGACG-3′), targeting an approximately 408 bp variable region (position 2135025–2134618 in the genome of *S. pneumoniae* TIGR4, GenBank accession no. AE005672.3) were designed to assess streptococcal species identification. This 408 bp sequence was validated using a reference dataset which we created from 501 S2-sequences from streptococcal species, extracted from whole-genome sequences available in the NCBI database (https://www.ncbi.nlm.nih.gov/genome) [2,52]. These reference S2-sequences were aligned using the MUSCLE algorithm in MEGA v6.0 [53] and phylogenetic analysis was performed using the minimum evolution method with nucleotide substitution type and Maximum Composite Likelihood Substitution Model with bootstrap analysis based on 500 replicates. The resulting tree was compared to that of whole-genome sequences published on the online PATRIC database of over 100 000 consistently annotated microbial genomes collected from GenBank and RefSeq [54]. Next, we used the S2 primers (10 µM each) in 25 µl reaction volumes with DreamTaq Master Mix (ThermoFisher Scientific, Landsmeer, the Netherlands) including 2.5 µl of a template (minimum 1 ng, average 70 ng of genomic DNA) to generate a PCR product for all 221 isolates included in the study. PCR conditions were as follows: 95°C for 15 min, then 40 cycles of 94°C for 30 s, 54°C for 1 min and 72°C for 1 min, followed by 60°C for 30 min. *S. pneumoniae* serotype 19A strain SJD86 was included as a positive control in each run [55]. Amplicons between 400 and 450 bp (approximately, because amplicons generated with the S2F-S2R primer pair can vary in size) were gel purified using the GeneJET PCR Purification kit (ThermoFisher Scientific), then 5 µl was mixed with the S2F (10 µM) or S2R (3 µM) primer and sequenced by Macrogen (Amsterdam, the Netherlands). Sequences generated were assembled using BioNumerics v5.10 (Applied Maths NV, http://www.applied-maths.com) and cross-referenced with the reference dataset for species annotation. Streptococcal strains included in the reference dataset and the S2-sequences (and accession numbers) of the study isolates are detailed in the electronic supplementary material, table S1. Strains within the Mitis group were designated according to the nomenclature proposed by Jensen *et al.* [5].

The sensitivity and specificity of S2-typing to discriminate streptococcal species was assessed using the 431 non-pneumococcal S2-sequences in the reference dataset and a total of 1227 pneumococcal S2-sequences (70 isolates in the reference dataset and 1157 pneumococcal meningitis isolates retrieved from the collection of the Netherlands Reference Laboratory for Bacterial Meningitis, NRLBM). Read data, assembled and annotated contigs of the 1157 pneumococcal meningitis isolates from NRLBM are deposited in the European Nucleotide Archive (ENA): study accession number PRJEB4909 (http://www.ebi.ac.uk/ena/data/view/PRJEB4909).

### 2.4. Detection of species-specific DNA sequences

All strains were tested in molecular assays targeting sequences (originally) reported to be unique for *S. pneumoniae* genes, namely *lytA* [26], *piaB* [24,27], Spn9802 [38], *cpsA* [47] and *ply* [48]. DNA of *S. pneumoniae* strain SJD86 was included as a positive control in every molecular assay [55]. In addition, all strains were tested for the presence of the pneumococcal virulence factor zinc metalloproteinase C gene, *zmpC*.

The presence of sequences matching pneumococcal genes *lytA* and *piaB* was assessed using previously described probe-based qPCRs [24,26,27]. The presence of Spn9802 [39] was assessed by qPCR using SYBRgreen chemistry (ThermoFisher Scientific) and primers described by Abdeldaim *et al.* [38]. Positivity for qPCR-signal was determined when C_T_ values matched 16S DNA concentrations. Conventional PCR (cPCR) was used to detect *ply* and *cpsA* (or *wzg*, a gene within the capsular polysaccharide biosynthesis operon) [47]. When reported, amplicons generated in cPCR were sequenced and analysed in SeqMan Pro (DNASTAR Lasergene v12.2.0, Madison, WI, USA) for homology to published sequences [34].

The presence of *zmpC,* was detected with cPCR using primers (ShortZmpC-F 5′-CAGCTGGTAACAGCCATGCAA-3′, ShortZmpC-R 5′-CAATGCACCATTTTCTAATCTACCD-3′) targeting a 563 bp fragment corresponding to position 75858–76420 bp in the genome sequence of *S. pneumoniae* strain TIGR4. One microlitre of DNA template (minimum 0.35 ng bacterial genomic DNA, average 28 ng) was tested with the ShortZmpC F-R primer pair (10 µM each) in a 12.5 µl reaction volume using DreamTaq Master Mix (ThermoFisher Scientific). PCR conditions were as described for the S2-typing PCR except for a 90 s annealing step (54°C). Amplicons (approx. 560 bp) were sequenced as described above. Strains which generated sequences 100% homologous to any published for *S. pneumoniae* were revisited with primers designed in *S. pneumoniae* to amplify the approximately 5000 bp [45] and approximately 8000 bp [46] fragments of *zmpC.* In 50 µl reaction volumes using GoTaq^®^ Long PCR Master Mix (Promega, Madison, USA), 4 µl of DNA template was tested with each primer pair (10 µM each). PCR conditions were as for the 560 bp assay but with longer steps at 72°C (5 min for 5000 bp, 8 min for 8000 bp products, per cycle). DNA of *S. pneumoniae* serotype 33F strain 2080133 was included as a positive control in all PCRs targeting *zmpC*.

### 2.5. Detection of pneumococcal capsule-type-specific DNA sequences

All strains were tested for pneumococcal serotype-specific signal in qPCR assays using primers and probes targeting serotypes/serogroups 1, 3, 6A/B/C/D, 7A/F, 8, 9A/N/V, 10A/B, 12A/B/F, 14, 15A/B/C, 19A, 20, 23F, 33A/F, 35B, 38 [29], 11A/D, 16F, 23A [50], 4, 5, 18B/C, 19F, 22A/F [29,50] and 35F [25]. The pneumococcal serotyping method of capsular sequence typing (CST) was also applied to all DNA templates [49]. For each serotype-specific qPCR, DNA of a clinical pneumococcal strain of the serotype(s) targeted was included as a positive control [34].

### 2.6. Conventional serotyping

Non-pneumococcal strains generating positive signals in serotype-specific qPCR assays were tested for the expression of capsular polysaccharides by the co-agglutination method of the Pneumotest kit [23] (Staten Serum Institut, SSI Diagnostica, Hillerød, Denmark) and by the Quellung method [22] using type-specific sera (Staten Serum Institut, SSI Diagnostica).

### 2.7. Statistics

Statistical analyses were conducted using GraphPad Prism v6.02 for Windows (GraphPad Software, CA, USA). Statistical significance was determined using Fisher's Exact test (unless otherwise stated) and defined as *p* < 0.05.

## 3. Results

### 3.1. Phylogenetic tree based on S2-sequences shows clustering according to streptococcal species

**Figure 1.**
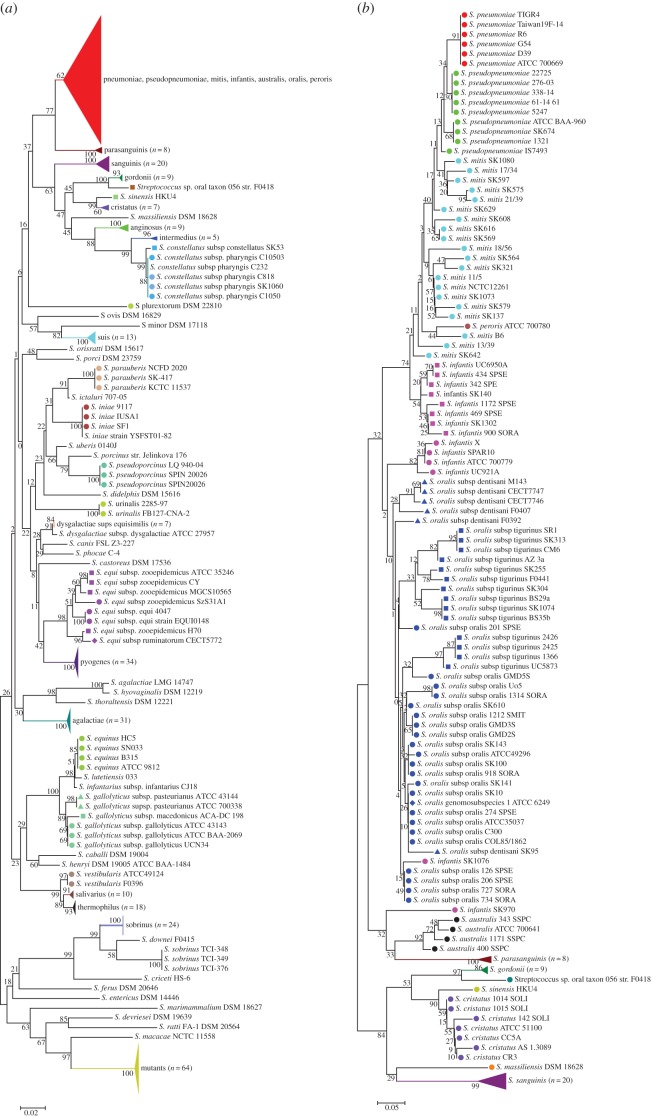
Cluster analyses of the S2-sequences of (*a*) the 498 streptococcal strains in the reference dataset (strains of streptococcal species pneumoniae, pseudopneumoniae, mitis, infantis, australis, oralis and peroris are collapsed together, red triangle) and (*b*) the 360 strains included in the study, belonging to the Mitis group. Phylogenetic analysis was performed using the minimum evolution method with nucleotide substitution type and Maximum Composite Likelihood Substitution Model with bootstrap analysis based on 500 replicates. Strains of the same species are grouped by colour. Different symbols of the same colour indicate subspecies. Magenta squares and circles indicate the two *S. infantis* clusters identified by Jensen *et al.* [5] (circles = cluster 1; squares = cluster 2). Different dark blue symbols indicate the different *S. oralis* subspecies identified by Jensen *et al.* [5].

Phylogenetic analysis of the streptococcal S2-reference dataset sequences showed grouping according to streptococcal species (figure 1*a*), comparable to the results from phylogenetic analyses of whole-genome sequences, which can be viewed on the PATRIC website (https://www.patricbrc.org/view/Taxonomy/1301#view_tab=phylogeny) [54].

Within the Mitis group, the S2-sequences of pneumococcal isolates clustered in a single clade with high reliability (figure 1*b*). Overall, S2-sequences of *S. pneumoniae* were very homogeneous. Examination of additional S2-sequences extracted from 1157 invasive *S. pneumoniae* whole genomes showed that except for one, all S2-sequences grouped together. The exception was an S2-sequence from a pneumococcal isolate, serotype 35B (2060880) from CSF, which was identical to that of *S. mitis* SK575. The blood isolate of the same patient had the same serotype and S2-type. Among the 1226 pneumococcal S2-sequences, excluding the one of isolate 2060880, only 11 polymorphic sites resulting in the same number of different alleles were found. Analyses of the complete *rpsB* sequence showed 19 polymorphisms unique to strain 2060880 and *S. mitis* SK575 dispersed over the entire gene.

Non-pneumococcal streptococci grouped into clusters according to newly proposed species nomenclature [5] but with deep branches. With the exception of SK970 and SK1076, *Streptococcus infantis* strains resolved into two clusters, according to *S. infantis* cluster 1 and cluster 2 observed by Jensen *et al*. [5] after phylogenetic analyses based on whole-genome sequences. *Streptococcus oralis* were distributed in two groups and did not group into the subspecies cluster observed with whole-genome analyses [5].

### 3.2. S2-typing of streptococcal strains from disease and carriage for species identification

Amplicons of expected size were generated for all streptococci tested in the study. None of the non-streptococcal strains yielded an S2-cPCR product. By cross-referencing the S2-sequences of the study isolates with the S2-sequences in the reference dataset, streptococcal species were clearly assigned to all 120 isolates from carriers and all 101 isolates cultured from disease. Of the 221 study isolates in total, 146 (66%) were classified as belonging to the Mitis group (32 isolates from disease, 114 isolates from carriage). This included the 17 *S. pneumoniae* strains (all from carriage and all Quellung non-typeable) which formed a distinct branch within the Mitis group cluster. Altogether, using S2-sequencing to discriminate *S. pneumoniae* from non-pneumococcal streptococci had a sensitivity of 99.92% (1226/1227) and a specificity of 100% (430/430). S2-sequences and species annotations are published under GenBank accessions MF375925–MF376145.

With differences in the selection criteria for clinical versus carriage isolates included in the study (clinical isolates included both α-haemolytic and β-haemolytic strains, whereas non-pneumococcal isolates from carriage were exclusively randomly selected α-haemolytic strains), we only tested for differences in the distribution of α-haemolytic non-pneumococcal streptococci in disease (*n* = 53) versus carriage (*n* = 103). We found *Streptococcus salivarius*, *Streptococcus sanguinis* and *Streptococcus gallolyticus* to be over-represented among strains of α-haemolytic streptococci from disease, whereas *S. infantis* and *S. mitis* to be over-represented among carriage isolates (table 2).

**Table 2.** Detection of pneumococcal-specific sequences in *Streptococcus* spp. strains, per S2-type. n.a., not applicable. Strains of this species were not included and tested in the current study. NT denotes all 17 *S. pneumoniae* strains were non-typeable. Significant difference (third column) in the proportion of α-haemolytic non-pneumococcal streptococci of the species cultured from disease (*n* = 53 total) versus carriage (*n* = 103 total), or (columns 4–10) in proportion of individual species strains positive for a particular molecular target when cultured from disease versus carriage.

streptococcal species according to S2-type	*n*	disease/carriage (n/n)	*lytA*	*piaB*	*ply*	*cps*	Spn9802	*zmpC* (560 bp)	*zmpC* (5 kb)	*zmpC* (8 kb)	qPCR serotype	CST serotype
**β-haemolytic streptococci**	**48**	**48/n.a.**										
*S. agalactiae*	9	9/n.a.	—^a^	—	—	—	—	—	—	—	—	—
*S. anginosus*	2	2/n.a.	—	—	—	—	—	—	—	—	—	—
*S. constellatus*	1	1/n.a.	—	—	—	—	—	—	—	—	—	—
*S. dysgalactiae* subsp*. equisimilis*	10	10/n.a.	—	—	1	—	—	—	—	—	—	—
*S. equi* subsp*. zooepidemicus*	2	2/n.a.	—	—	—	—	—	1	—	—	—	—
*S. intermedius*	4	4/n.a.	—	—	—	—	—	—	—	—	—	—
*S. pyogenes*	10	10/n.a.	—	—	—	—	—	—	—	—	—	—
*S. suis*	10	10/n.a.	—	—	—	—	—	—	—	—	—	—
**α-haemolytic streptococci**	**174**	**53/103**										
*S. australis*	2	0/2	—	—	—	—	—	—	—	—	—	—
*S. cristatus*	3	2/1	—	—	—	—	—	—	—	—	—	—
*S. gallolyticus* subsp*. Gallolyticus*^c^	2	2/0	—	—	—	—	—	—	—	—	—	—
*S. gallolyticus* subsp*. Pasteurianus*^c^	2	2/0	—	—	—	—	—	—	—	—	—	—
*S. gordonii*	1	1/0	—	—	—	—	—	—	—	—	—	—
*S. infantis*	22	3/19*	—	—	—	—	—	0/3^b^	0/1	—	0/1	—
*S. mitis*	68	13/56***	—	—	5/13	—	—	0/2	0/1	—	1/3	—
*S. oralis*	25	10/15	—	—	—	1/0	1/0	0/1	—	—	—	—
*S. parasanguinis*	7	0/7	—	—	—	—	—	—	—	—	—	—
*S. pseudopneumoniae*	6	3/3	—	—	2/1	—	3/0	—	—	—	—	—
*S. salivarius*	12	11/1^#^	—	—	—	—	—	—	—	—	—	—
*S. sanguinis*	7	6/1**	—	—	—	—	—	—	—	—	—	—
*S. pneumoniae^NT^*	17	n.a./17	17	3	15	3	7	10	10	—	3	3
total	221	101/120	0/17	0/3	8/29	1/3	4/7	1/16	0/12	0/0	1/7	0/3

^a^None of the strains tested of the particular species generated an amplicon with 100% sequence homology to sequences published for *S. pneumoniae.*

^b^Strains which generated amplicons with 100% sequence homology to sequences published for *S. pneumoniae*.

^c^Also *γ-*haemolytic.

*p < 0.05, **p < 0.01, ***p < 0.001 ^#^*p* < 0.0001 (Fisher's exact test).

### 3.3. Distribution of pneumococcal-specific genes among non-pneumococcal streptococci

All 221 streptococcal and nine non-streptococcal isolates were assessed in molecular assays used to detect *S. pneumoniae*. Results according to S2-type are detailed in table 2 (summarized for disease versus carriage isolates in the electronic supplementary material, table S2). Individual strains testing positive for any molecular target are detailed further in table 3. All pneumococcus-specific PCRs remained negative when the non-streptococcal strains were tested.

**Table 3.** Detailed results of non-pneumococcal streptococcal strains generating false positive signals when tested in molecular assays for common pneumococcal molecular targets.

	*lytA*	*piaB*	*ply*	*cpsA*	Spn9802	*zmpC*^b^ (560 bp)	*zmpC* (5 kb)	*zmpC* (8 kb)	qPCR serotype
**strains from invasive disease^a^**									
*S. dysgalactiae* subsp. *equisimilis*			+						
*S. equi* subsp*. zooepidemicus*						+			
*S. mitis* (*n* = 4)			+						
*S. mitis*			+						19F
*S. oralis*					+				
*S. oralis*				+					
*S. pseudopneumoniae*					+				
*S. pseudopneumoniae* (*n* = 2)			+		+				
**strains from asymptomatic carriage^a^**									
*S. infantis*									9A/N/V
*S. infantis*						+	+		
*S. infantis* (*n* = 2)						+			
*S. mitis*									5
*S. mitis*									18B/C
*S. mitis*									19F
*S. mitis*			+			+			
*S. mitis*			+			+	+		
*S. mitis* (10 strains)			+						
*S. oralis*						+			
*S. pseudopneumoniae*			+						

^a^Strains annotated based on sequence analysis of the ribosomal S2 gene.

^b^Strains which generated PCR amplicons with full homology to sequence published for *S. pneumoniae*.

All 204 non-pneumococcal isolates were negative in *lytA*- and *piaB-*specific qPCRs, yet 4 (2%) were positive for Spn9802. cPCRs targeting *cpsA* and *ply* yielded amplicons of correct size in one (0.5%) and 22 (11%) isolates, respectively. None of the *cpsA* or *ply* amplicons were of full homology to any sequence published for *S. pneumoniae*.

Twelve non-pneumococcal streptococci yielded amplicons of expected size in the 560 bp *zmpC*-specific cPCR. Following amplicon-sequencing, seven isolates (disease: *Streptococcus equi subsp. zooepidemicus* H70, *n* = 1; carriage: *S. mitis*, *n* = 2; *S. infantis*, *n* = 3; *S. oralis*, *n* = 1) were 100% homologous to the nucleotide sequences of unencapsulated *S. pneumoniae* strain NT_110_58 (GenBank accession no. CP007593.1) and encapsulated strains of serotypes 19F (CP001015.1) and 11A (CP001015.1). Of the seven isolates positive for the 563 bp *zmpC* amplicons with sequence homologous to that reported in *S. pneumoniae*, one *S. mitis* and one *S. infantis* strain also generated product in the 5000 bp cPCR of size reported for some non-typeable *S. pneumoniae* [45]. None of the 221 study isolates was positive for the 8000 bp cPCR product reported for encapsulated pneumococci, demonstrating better specificity of this cPCR for detecting pneumococcal-specific *zmpC* sequences [46]. Of note, among α-haemolytic non-pneumococcal streptococcal strains the confounding results for *zmpC* were observed exclusively among isolates from carriers (*n* = 6 of 103 strains from carriage versus none of 53 clinical isolates, *p* = 0.096; table 2).

### 3.4. Distribution of pneumococcal-specific genes among non-typeable pneumococci

All 17 pneumococci non-typeable by Quellung were *lytA*-positive*,* yet only three (18%) were *piaB*-positive (table 4). This was consistent with previous reports from us [27] and others [43] but also with the *piaB* gene being absent in published sequences of non-typeable pneumococci (https://www.ncbi.nlm.nih.gov/genome). The three *piaB*-positive non-typeable pneumococci were also the only unencapsulated strains *cpsA-*positive by cPCR*.* When tested in the *ply-*cPCR however, 15 (88%) non-typeable isolates produced amplicons of expected size. None of the *cpsA* or *ply* amplicons were of full homology to any sequence published for *S. pneumoniae*. In addition, six (35%) non-typeable isolates generated sequence-specific signal in the Spn9802-qPCR.

**Table 4.** *Streptococcus pneumoniae* strains non-typeable (thus, unencapsulated) by the conventional diagnostic method, positive when tested in molecular assays for common pneumococcal molecular targets.

number of isolates	*lytA*	*piaB*	*ply*	*cpsA*	Spn9802	*zmpC***^a^** (560 bp)	*zmpC* (5 kb)	*zmpC* (8 kb)	qPCR serotype	CST serotype
9	+		+			+	+			
3	+		+		+					
1	+		+		+	+	+			
1	+									
1	+	+	+	+	+				22A/F	22F
1	+	+	+	+	+					25F
1	+	+		+	+				19A	19A
total n (*%*)	17 (*100*)	3 (*18*)	15 (*90*)	3 (*18*)	7 (*40*)	10 (*59*)	10 (*59*)	0 (*0*)	2 (*12*)	3 (*18*)

^a^Strains which generated PCR amplicons with full homology to sequences published for *S. pneumoniae*.

When tested for *zmpC*, 10 non-typeable strains (59% of 17) produced size-specific amplicon in the 560 bp cPCR which following sequencing were all 100% homologous to the published sequences for the *S. pneumoniae* strains detailed above. Subsequently, all 10 also produced size-specific amplicons in the 5000 bp cPCR, but none in the 8000 bp cPCR.

### 3.5. Detection of serotype-specific sequences and capsular polysaccharides

Of 204 non-pneumococcal streptococcal isolates, 42 (21%) yielded amplicons of expected size in CST-cPCR [49]. However, all sequences of these amplicons showed less than 75% sequence homology to those reported for *S. pneumoniae* and CST results were therefore regarded as negative.

Five non-pneumococcal streptococci (2% of 204 isolates) yielded a serotype-specific signal in pneumococcal serotype/serogroup-specific qPCRs (tables 3 and 4) [28,34]. Of disease strains, a single isolate S2-typed as *S. mitis* (also *ply*-positive in cPCR) generated serotype-specific signal in the 19F-qPCR assay published by Carvalho *et al.* [29]. Among carriage isolates, serotype-specific signals were yielded in qPCR assays published by the same authors and targeting serotypes/serogroups 5, 18B/C, 19F [29] (three isolates S2-typed as *S. mitis*, each positive in a single serotyping-qPCR assay) and 9A/N/V [29] (single isolate S2-typed as *S. infantis*). None of the isolates yielded a positive result in any assay published by Pimenta *et al.* [50]. Of the five non-pneumococcal strains yielding positive signals in serotyping-qPCR assays, two were also positive for the corresponding capsular type (serotype 5 and serogroup 9) in the co-agglutination test. However, none was typeable by the Quellung method. None of the nine non-streptococcal isolates yielded a serotype-specific signal in any of the genotyping assays.

Of the 17 Quellung non-typeable pneumococci from carriage, three (18%) were CST-positive (one strain positive for each of the serotypes 19A, 22F and 25F). All three were also *piaB*- and *cpsA*-positive. From these three strains, CST-positivity for serotypes 19A and 22A/F was in agreement with our panel of qPCR serotyping assays (an assay for detection of serotype 25F is not available within this qPCR panel).

## 4. Discussion

Quicker and more accurate diagnostic methods of pathogen detection advance treatment of infection and contribute to our understanding of disease aetiology [25,35,47,55,56]. Molecular-based diagnostic methods continue to evolve, improving detection of aetiological agents causing streptococcal disease. This also contributes to advances in surveillance of disease and carriage of the clinically most relevant streptococcal species, *S. pneumoniae* [25,35,57]. This is of particular importance following the introduction of commercial vaccines targeting pneumococcal disease, with molecular method-based surveillance studies already being implemented to monitor vaccine effects in disease and in carriage [58–61]. We demonstrate, however, that this is not without its challenges. Our current study highlights important considerations for the transition from conventional to molecular diagnostic methods. We showed that S2-sequencing discriminated *S. pneumoniae* from non-pneumococcal streptococci with high sensitivity (greater than 99%) and specificity (100%).

Jolley *et al*. [44] recently demonstrated that streptococci grouped according to species in phylogenetic analyses using sequences of all ribosomal protein genes. In this study, we classified species of streptococcal strains through sequencing of a variable region of the ribosomal S2 gene. The resulting trees (figure 1*a*,*b*) do not completely follow the topology of the trees based on whole-genome sequences, likely due to the much smaller S2-sequences used in our study [5,44]. However, the deviations observed are not of relevance, because S2-sequencing is not intended for studying evolution, but rather for use as a potent and fast tool for streptococcal species identification.

Outside the Mitis group strains grouped accordingly, with the exception of three *Streptococcus sobrinus* strains which formed a branch separated from the other 24 *S. sobrinus* strains and *Streptococcus agalactiae* LMG14747 grouped together with *Streptococcus hyovaginalis* DSM12219 but apart from the other 32 *S. agalactiae*. Analyses based on whole-genome sequences are needed to determine whether these strains are genuine *S. sobrinus* and *S. agalactiae*, respectively, or have to be reclassified. Within the Mitis group, *S. pneumoniae* could be clearly and robustly distinguished from non-pneumococcal strains. *S. sanguinis*, *Streptococcus parasanguinis*, *Streptococcus cristatus*, *Streptococcus gordonii* and the two clusters of *S. infantis* (with the exception of two strains) also grouped with high fidelity. *S. pseudopneumoniae*, *S. mitis* and *S. oralis* also formed distinct clusters but with low fidelity consistent with the deep branching of the *S. mitis* and *S. oralis* strains in whole-genome sequences and the close relatedness between these species and between *S. pseudopneumoniae* to both *S. mitis* and *S. pneumoniae* [1,2,5,7,62]. Ultimately, these observations exemplify the difficulty in streptococcal species annotation through biochemical (immunological) and genetic identification among streptococci colonizing the human upper respiratory tract and oral cavity. This arises from frequent HDT among Mitis group streptococci, which includes genes and their products targeted by diagnostic tests [2,11].

Clear examples of this were evident in our study. Among the 1227 S2-sequences from pneumococci, we observed one with a *S. mitis* type. This CSF isolate with serotype 35B and ST558 from a meningitis patient was a genuine pneumococcal isolate according to *in silico* DNA–DNA hybridization values (electronic supplementary material, data) and was *lytA* and *piaB*-positive. In addition, the isolate from the blood of the same patient tested positive for the identical S2-sequence and serotype. Close examination of the nucleotide sequence flanking *rpsB* in the whole-genome sequence of the CSF isolate showed a sequence of approximately 1 kbp upstream of *rpsB*, comprising an open reading frame (ORF) putatively encoding an amidase, with the highest nucleotide identities to the sequence in *S. mitis* strain SVGS_061 (95%), while the nucleotide identities with the sequence in *S. pneumoniae* strains was lower (87%). In addition, we saw evidence of HDT in non-pneumococcal streptococcal species testing positive for sequences of *cpsA*, *ply*, *zmpC* and capsular genes, all previously regarded as unique for *S. pneumoniae* and for products of these genes, namely pneumococcal capsular polysaccharides detected with the co-agglutination method. The detection of these genes among non-pneumococcal Mitis group streptococci highlights that caution must be taken when interpreting PCR results in assays targeting *cpsA*, *ply*, Spn9802 and *zmpC* when applied to polymicrobial samples and/or samples culture-negative for *S. pneumoniae*; positive signal may in fact represent confounders which could skew results of disease and carriage surveillance.

Interestingly, non-pneumococcal strains harbouring homologues of genes coding for *S. pneumoniae* virulence factors have been reported as more commonly associated with disease isolates when compared with carriage isolates of the corresponding species [1]. This does not seem to be the case for the sequences targeted in molecular diagnostic assays in our study. Although none of the confounding results was unique for streptococcal strains cultured from either disease or carriers, ‘false positivity’ was more common among the species of α-haemolytic streptococci that were over-represented in carriage—*S. infantis* and *S. mitis* in particular.

There is always a potential that genetic exchange will impede identification based on single targets as compared to identification based on whole-genome sequencing. However, considering the strength of species grouping by the S2-typing method introduced here, we propose the much simpler and more time-efficient S2-typing for use in reference laboratories for the identification of streptococcal species isolated from disease, particularly for the distinction of pneumococci from streptococcal strains confounding pneumococcal diagnostic tests. In addition, with adaptation to a deep-sequencing format, S2-typing could improve the annotation of streptococcal species in microbiome studies currently being based on the less discriminatory 16S gene sequencing, particularly in studies of the respiratory or oral microbiomes [63].

For the analysis of polymicrobial samples, molecular assays targeting specific DNA sequences increase the sensitivity of detection when compared with culture-based methods [25,27–30,34]. For surveillance on pneumococcal carriage, the higher sensitivity of molecular methods for detecting pneumococci can often only be inferred from samples from which live pneumococci cannot be isolated. Therefore, it is essential that assay specificity is carefully assessed. For the molecular detection of pneumococci, the *lytA*-qPCR assay is fast becoming the standard. However, with *lytA* homologues in non-pneumococcal streptococci and on prophages [1,17,19,20,35,38,43,64] and with one recent report of a *S. pseudopneumoniae* strain testing positive in the *lytA*-specific qPCR tested in this study [56], targeting a second pneumococcal-specific gene in polymicrobial samples reduces the likelihood of misclassification due to false positivity—the chance that confounding bacteria would acquire two genetic markers is low. Given the high specificity observed in the current study and the high concordance between qPCR results and the presence of live pneumococci in samples from children, adults and the elderly [27,28,34], we and others [43] recommend *piaB* as a suitable countermark to the *lytA*-qPCR for pneumococcal detection. The *piaB* distribution in streptococcal strains reported here is in line with results published by us [27] and others [43,65] showing *piaB* being unique for *S. pneumoniae* yet absent exclusively from non-typeable pneumococci. Interestingly, because *piaB*-negative non-typeable pneumococci are absent from IPD but not from carriage, acquired immune responses specific to Pia proteins [66,67] could potentially not only protect against disease by Pia-positive, presumably encapsulated pneumococci, but also increase the fitness costs for such strains competing within the respiratory niche, thus promoting carriage of less virulent non-typeable pneumococci.

Pneumococcal serotyping is also progressively transitioning from a reliance on phenotypic serological methods, such as Quellung and co-agglutination assays, to genotypic methods of serotype determination [25,49,68,69]. Here, we demonstrate the specificity of CST for serotyping pneumococcal isolates [70]. While targeting other serotype-specific genes has demonstrated a lack of specificity due to homologous sequences in non-pneumococcal Mitis group streptococci [11,71], sequencing a *wzh* gene fragment of the capsular locus was highly specific, supporting its potential as a reliable alternative to culture-dependent pneumococcal serotyping or to molecular methods requiring multiple assays. However, despite the specificity of molecular methods when applied to pure pneumococcal isolates [25,49,68], their reliability when applied to polymicrobial samples must be carefully monitored, due to reports of false positive signals from non-pneumococcal species [24,33,34,42]. In this study, comparatively few non-pneumococcal strains generated signal in serotype-specific assays. This implies that the validation of any serotype-specific assay should not only include testing pneumococcal strains of other serotypes and non-pneumococcal strains, but should also include testing of polymicrobial samples negative for pneumococcus-specific signal.

It should also be stressed that phenotypic methods for pneumococcal serotyping are also not exempt from confounding by (non)pneumococcal strains producing atypical reactions [72,73]. Here, we detected false positivity in the co-agglutination test used to determine type of pneumococcal capsular polysaccharide present in a sample, presumably through the presence of antigenic determinants common to those of *S. pneumoniae* [11,74,75]. While this is not a new observation [11,20], it has important implications for diagnostic strategies developed on this immunochemistry.

Owing to the importance of capsule for pneumococcal virulence and vaccination strategies, historically, studies of unencapsulated (non-typeable) pneumococci seldom progressed beyond identification as *S. pneumoniae* [1]. Non-typeable pneumococci are being increasingly detected in carriage surveillance following PCV implementation [76,77], an important consideration due to their higher rates of recombination [78] and greater number of mobile elements [79] than encapsulated strains. However, their prevalence may be skewed when grouped with non-pneumococcal confounders of culture-based methods, or if overlooked due to their atypical phenotype on culture plates [79]. Here, all non-typeable pneumococci were convincingly S2-typed as *S. pneumoniae*. Despite a lack of capsule, unencapsulated pneumococci have been shown to colonize the nasopharynx of mice as efficiently as encapsulated strains [73,77] and are disproportionately identified as the aetiological agent of highly contagious pneumococcal conjunctivitis [80]. One such gene that might play a role in this is *zmpC*, suggested to have been only recently acquired by pneumococci [81] and present in only a limited number of strains [46,69]. While studies have previously speculated a role for ZmpC in invasive disease [46,69], it has recently been demonstrated that ZmpC suppresses *S. pneumoniae* virulence in experimental models of pneumococcal meningitis [82]. Owing to its prevalence in non-typeable pneumococci in the current study (strains which are seldom isolated from invasive disease [9]) and its association with increased adhesion to host mucosal cells [46], our findings further support a role for ZmpC in colonization rather than in pneumococcal disease [81].

In conclusion, in the current study we further demonstrate the potential for misidentification of streptococci, usually carried as harmless respiratory commensals, but with the ability to cause severe disease. While we target the most pathogenic of these—*S. pneumoniae*—with vaccination programmes, accurate species identification is crucial for the reliable monitoring of pneumococcal disease and effects of vaccination strategies. Conventional diagnostic methods are insensitive in both disease diagnosis [29,56] and in carriage studies [24,28,34]. New methods are required but carry a risk of over-detection or misidentification if subject to confounding from co-occurring species. Here, we employed S2-typing to identify streptococci which may confound both phenotypic and genotypic methods of pneumococcal detection and serotype determination [17,83]. We propose S2-typing for use in reference laboratories to assist in species annotation of streptococcal strains and for the classification of *S. pneumoniae* reliably from strains confounding pneumococcal diagnostic tests. Furthermore, S2-typing provides a sensitive method to distinguish non-typeable pneumococcal strains from other streptococci generating atypical reactions and to identify individual species contributing genes coding virulence factors to the genetic pool [73]. For enhanced detection of pneumococci in carriage surveillance studies using polymicrobial samples, to increase sensitivity of pneumococcal detection our findings support the use of qPCR assays targeting species-specific regions of the genes *lytA* and *piaB*.

## Supplementary Material

Supplementary Table S1

## Supplementary Material

Supplementary Table S2

## Supplementary Material

Supplementary Data
