# Supplementary material for: Sequencing of the variable region of rpsB to discriminate between Streptococcus pneumoniae and other streptococcal species
Source: Open Biol. 2017 Sep 20;7(9):170074. doi: 10.1098/rsob.170074 (PMC5627049; doi:10.1098/rsob.170074)
Supplement: Supplementary Table S2 [file rsob170074supp2.pdf]

Supplementary Table S2. Number (%) of non-pneumococcal streptococcal strains positive when tested in molecular assays for common pneumococcal molecular targets.

**Source of strain**[a]	**all**	***lytA***	***piaB***	***ply***	***cpsA***	**Spn9802**	***zmpC***[b] **(500 bp)**	***zmpC*** **(5kb bp)**	***zmpC*** **(8kb bp)**	**qPCR serotype**	**CST serotype**
**Disease**	101	0	0	8 (8)	1 (1)	4 (4)	1 (1)	0	0	1 (1)	0
**Carriage**	103	0	0	14 (14)	0	0	6 (6)	2 (2)	0	4 (4)	0
**Overall**	**204**	**0**	**0**	**22 (10)**	**1 (0.5)**	**4 (2)**	**7 (3)**	**2 (1)**	**0**	**5 (2)**	**0**

[a]strains annotated as non-pneumococci based on sequence analysis of the ribosomal S2 gene.

[b]strains which generated amplicons with 100% sequence homology to published sequences for *S. pneumoniae.*
