# Supplementary material for: Sequencing of the variable region of rpsB to discriminate between Streptococcus pneumoniae and other streptococcal species
Source: Open Biol. 2017 Sep 20;7(9):170074. doi: 10.1098/rsob.170074 (PMC5627049; doi:10.1098/rsob.170074)
Supplement: Supplementary Data [file rsob170074supp3.pdf]

In the current study, examination of 1157 S2 sequences extracted from invasive *Streptococcus pneumoniae* whole genomes revealed that except for one, all S2-sequences grouped together. The exception was an S2-sequence from a pneumococcal isolate serotype 35B (2060880) from CSF, which was identical to that of *S. mitis* SK575. The blood isolate of the same patient had the same serotype and S2-type. To confirm the species of this strain, we performed *in silico* DNA hybridisation using the recommended formula and the Genome-To-Genome Distance Calculator (http://ggdc.dsmz.de/distcalc2.php) (1).

***DNA-DNA hybridisation (DDH) estimate (in silico) (GLM-based) (identities / High-scoring Segment Pair (HSP) length)***

| **Strain** | **2060880** | **2070268** | **2100466** | **2120989** | ***S. mitis* SK575** |
|---|---|---|---|---|---|
| ***S. pneumoniae* TIGR4** | 87.30% [84.7 - 89.5%] | 87.40% [84.9 - 89.6%] | 87.40% [84.8 - 89.6%] | 87.70% [85.1 - 89.8%] | 47.80% [45.2 - 50.4%] |
| ***S. pneumoniae* D39** | 92.90% [90.1 - 94.9%] | 88.80% [86.3 - 90.8%] | 88.70% [86.3 - 90.8%] | 88.90% [86.5 - 90.9%] | 43.00% [40.5 - 45.6%] |
| ***S. pneumoniae* 35B 2060880** | 100.00% [100 - NaN%] | 88.90% [86.4 - 90.9%] | 88.70% [86.3 - 90.8%] | 89.00% [86.6 - 91%] | 39.50% [37.1 - 42.1%] |
| ***S. pneumoniae* 35B 2070268** | | 100.00% [100 - NaN%] | 99.00% [98.5 - 99.4%] | 99.40% [99 - 99.6%] | 47.20% [44.6 - 49.8%] |
| ***S. pneumoniae* 35B 2100466** | | | 100.00% [100 - NaN%] | 99.10% [98.6 - 99.4%] | 47.00% [44.5 - 49.6%] |
| ***S. pneumoniae* 35B 2120989** | | | | 100.00% [100- %] | 47.00% [44.5 - 49.6%] |
| ***S. mitis* SK575** | | | | | 100.00% [100 - %] |

***Probability of being the same species (identities / HSP length)***

| **Strain** | **2060880** | **2070268** | **2100466** | **2120989** | ***S. mitis* SK575** |
|---|---|---|---|---|---|
| ***S. pneumoniae* TIGR4** | 94.78% | 94.84% | 94.81% | 94.93% | 13.54% |
| ***S. pneumoniae* D39** | 98.16% | 95.36% | 95.34% | 95.41% | 5.47% |
| ***S. pneumoniae* 35B 2060880** | 98.30% | 95.39% | 95.34% | 95.45% | 2.41% |
| ***S. pneumoniae* 35B 2070268** | | 99.60% | 98.07% | 98.14% | 27.20% |
| ***S. pneumoniae* 35B 2100466** | | | 98.30% | 98.08% | 11.94% |
| ***S. pneumoniae* 35B 2120989** | | | | 98.30% | 11.95% |
| ***S. mitis* SK575** | | | | | 98.30% |

**Reference**


1. Meier-Kolthoff JP, Auch AF, Klenk H-P, Göker M. Genome sequence-based species delimitation with confidence intervals and improved distance functions. BMC Bioinformatics. 2013;14(1):60.
